# Does pomegranate extract supplementation improve the clinical symptoms of patients with allergic asthma? A double-blind, randomized, placebo-controlled trial

**DOI:** 10.3389/fphar.2023.1109966

**Published:** 2023-01-25

**Authors:** Seyed Ahmad Hosseini, Zainab Shateri, Farhad Abolnezhadian, Elham Maraghi, Maryam Haddadzadeh Shoushtari, Marzie Zilaee

**Affiliations:** ^1^ Nutrition and Metabolic Diseases Research Center, Clinical Research Institute, Ahvaz Jundishapur University of Medical Sciences, Ahvaz, Iran; ^2^ Student Research Committee, Ahvaz Jundishapur University of Medical Sciences, Ahvaz, Iran; ^3^ Division of Immunology and Allergy, Department of Pediatrics, Abuzar Children’s Hospital, Ahvaz Jundishapur University of Medical Sciences, Ahvaz, Iran; ^4^ Department of Biostatistics and Epidemiology, School of Public Health, Ahvaz Jundishapur University of Medical Sciences, Ahvaz, Iran; ^5^ Air Pollution and Respiratory Disease Research Center, Ahvaz Jundishapur University of Medical Sciences, Ahvaz, Iran

**Keywords:** allergic asthma, blood cell count, complete blood count, CBC, clinical symptoms, clinical trial, pomegranate, Punica granatum

## Abstract

**Background:** Asthma essentially represents a chronic inflammatory disease that manifests as a lifelong condition with different severity throughout the life of patients with asthma. Pomegranate holds three times the antioxidant activity compared to other polyphenol-rich food sources like green tea, which may positively impact asthma.

**Aim of the study:** This research aimed to investigate the pomegranate supplementation influences clinical symptoms, eosinophil, basophil, and neutrophil counts in patients with allergic asthma.

**Materials and Methods:** Participants (*n* = 64) suffering from mild to moderate allergic asthma were randomly divided into two groups: The control group received placebo capsules and the intervention group received 250 mg pomegranate extract capsules twice a day (for 8 weeks). To analyze the data, we used SPSS software (version 22). The significance level of *p*-value was considered less than 0.05.

**Results:** The findings showed that the pomegranate extract improved patients’ clinical symptoms like daily breath shortness, nocturnal breath shortness, and limitation of asthma-related activity in the intervention group compared to the control group. Furthermore, eosinophil, basophil, and neutrophil counts were significantly decreased in the intervention group. Also, by comparing the two groups, the levels of change in neutrophils and eosinophils were statistically significant.

**Conclusion:** It appears that the pomegranate extract can ameliorate some clinical symptoms and reduce neutrophils, basophils, and eosinophils in allergic asthma patients.

**Clinical Trial Registration:** https://www.irct.ir/trial/45612; identifier: IRCT20200205046384N1.

## 1 Introduction

Asthma represents a chronic inflammatory disease that manifests as a lifelong condition with different severity throughout the life of patients with asthma (Nunes et al., 2017). In fact, it is a complex, multifactorial, chronic disease related to the respiratory tract associated with inflammation of the airways, which increases reaction and regeneration in response to other types of physical and chemical stimuli (Kumari et al., 2015).

Research has shown the most common asthma symptoms include airway obstruction or reversibility of obstruction, wheezing, and breath shortness (Gauthier et al., 2015). Asthma symptoms may be intermittent or persistent, displaying as mild, moderate, or severe (Bateman et al., 2008).

In allergic asthma, increased immunoglobulin E (IgE) production in response to environmental allergens represents the most substantial detectable factor for asthma progression, mainly when allergies occur in the early stages of life (Hamelmann, 2007).

According to published statistics, more than 15 million people have asthma yearly (Hasankhani et al., 2013). It is estimated that there are currently 300 million asthma patients worldwide; by 2025, 100 million people might be added to this amount. It has been demonstrated that the prevalence of asthma in adults is 4.3% worldwide (To et al., 2012). According to the National Health Information, the prevalence of asthma in adults aged 20 to 44 in Iran was reported to be 8.9%, which shows an increasing prevalence over the last decade (Fazlollahi et al., 2018). The prevalence of asthma is reported between 5% and 15% in Iran (Hasankhani et al., 2013). The number of people with asthma in Ahvaz is more than the average of the whole of Iran (Raji et al., 2020). Recent research was shown poor control of asthma exists in 53%–58% of patients despite receiving appropriate treatment (Hasankhani et al., 2013), which can justify the high prevalence of asthma disease.

The pomegranate is an edible fruit cultivated in many countries, including Iran (Aviram et al., 2000), the most significant producer of pomegranate in the world (Chandra et al., 2010). The edible part of a pomegranate (approximately 50% of the total weight of the fruit) contains 80% water and 20% seeds (Raji et al., 2020).

In traditional medicine, pomegranate has been used to treat diseases due to its bioactive compounds. It has anti-hepatotoxic, anti-diabetic, anti-tumor, antimicrobial, anti-inflammatory, and anti-viral properties, influencing skin, oral and cardiovascular conditions (Rouhi et al., 2017). The pomegranate is considered a rich source of polyphenols (Mertens-Talcott et al., 2006). It has been indicated that polyphenols can retain antioxidant and anti-inflammatory features in the human body. In addition, it has been shown that pomegranate holds three times the antioxidant activity compared to other polyphenol-rich food sources like green tea (Danesi and Ferguson, 2017).

In asthma, there is continuous airway inflammation with a mucosal influx of T lymphocytes, eosinophils, mast cells, and the release of proinflammatory cytokines and mediators of lipids (Barnig et al., 2018). Eosinophils and neutrophils are the primary cells involved in asthma disease pathology and inflammation (Bloemen et al., 2007; Chandra et al., 2010; Pelaia et al., 2015).

Rogerio et al. evaluated the effect of one of the active ingredients of pomegranate called ellagic acid in mice with asthma. It was found that ellagic acid reduced the number of eosinophils and neutrophils (Rogerio et al., 2008). Also, a study conducted by Bachoual et al. was demonstrated that the extract of pomegranate peel inhibited myeloperoxidase of neutrophils *in vitro* and reduced pneumonia (Bachoual et al., 2011). Furthermore, Alves et al. assessed the effect of ellagic acid on allergic airway response in mice with asthma. It was shown that ellagic acid accelerates airway clearance by reducing total leukocytes and eosinophils (de Freitas Alves et al., 2013). 

Corticosteroids like dexamethasone are currently used as anti-asthmatic. Still, various studies have shown that microbes in the airways can inhibit corticosteroid reactions and potentially affect corticosteroid therapy’s effectiveness in treating asthma (Kumari et al., 2015). Hence, based on the antioxidant and anti-inflammatory features of the pomegranate, it appears pomegranate extract administration can helpfully affect patients with asthma. In contrast, the effect of pomegranate extract on allergic asthma has not been studied in human models. Therefore, this investigation aimed to study the effects of pomegranate extract on complete blood count with differential (CBC-diff) and clinical symptoms in patients with allergic asthma (mild to moderate severity).

## 2 Materials and methods

The current study used a double-blind randomized controlled trial. Participants were people with allergic asthma who attended the allergy and asthma center. Using the average comparison formula with *β* = 0.2, *α* = 0.05, and S = 0.7 based on the previous study, (Zilaee et al., 2019) the sample size was computed for the serum levels of eosinophils. Therefore, at least 31 people were required to participate in each group. Assuming 10% violation of protocols or removal, we allocated 35 patients to each group. The individuals in the present study were ultimately assigned to each group based on the severity of the disease (1:1 ratio) and clinical symptoms according to a permuted block randomization (set by a biostatistician). In order to perform a double-blind study, one person was requested to number the capsule bottles.

After explaining the method to the participants, blood samples were taken to check the serum IgE level to determine allergic asthma (Bateman et al., 2008; Zilaee et al., 2019). Since serum IgE levels are more valid for confirming allergic asthma compared to other tests (such as skin prick test), we applied IgE serum levels to determine allergic asthma (Sunyer et al., 1995). According to previous studies, serum IgE level ≥30 international units (IU) are considered allergic asthma (Bateman et al., 2008; Mason et al., 2015). Therefore, patients with IgE ≥30 IU were studied. At the beginning of the investigation, the consent form was taken from the participants, and then they were requested to complete the research questionnaires. Questionnaires were filled again at the end of the study. Information on the patients’ clinical symptoms was asked, including daily breath shortness, nocturnal breath shortness, limitation of asthma-related activity, salbutamol spray usage, and nocturnal waking up.

One of the valuable tools to evaluate lung function is the spirometry test (Sewa and Ong, 2014). Spirometry is one of the tests of pulmonary function to assess clinical symptoms and the response of patients with asthma to drugs. Forced expiratory volume in 1 s (FEV1) is one of the most important parameters of the spirometry test (Bateman et al., 2008). FEV_1_ correlates with the severity of airway obstruction and is the most reproducible lung function parameter (Enright et al., 2012). Patients with allergic asthma were divided into two groups (moderate and mild) using clinical symptoms applying the Global Initiative for Asthma (GINA) diagnostic criteria and its severity (FEV_1_ ≥ 60% by spirometry test). In mild persistent asthma, symptoms occur less than once a day and more than once a week. The disease symptoms may influence the patient’s sleep and activity. FEV_1_ is ≥80% and asthma symptoms are experienced at night more than twice a month. In moderate persistent asthma, symptoms occur daily and may influence the patient’s sleep and activity. FEV_1_ is 60%–80% and asthma symptoms occur at night more than once a week (Bateman et al., 2008).

A permutated block randomization method was used to randomly allocate subjects into the supplement and placebo groups, and their consumption and possible side effects were monitored weekly by phone call. Supplements were given to individuals in two stages (in the 1^st^ and 4^th^ week). Patients were asked to stop consuming the pomegranate, pomegranate paste, pomegranate juice, and products containing pomegranate during the research period. It was also requested that the capsule bottles be delivered at the end of the study. Individuals who did not regularly consume the capsules or were sensitive to the supplements were excluded from the study. Anthropometric measurements, including waist circumference (WC), weight, hip circumference, and waist-to-hip ratio (WHR), were measured at the study’s beginning and end. Height was also recorded at the beginning of the research.

Duration of asthma disease, demographic information, history of medication use, level of physical activity using the short-form of international physical activity questionnaire, and blood pressure was evaluated at the beginning and the end of the study.

The researchers, the physician, and all the participants followed a double-blinded scheme using a numbering method for the bottles until the completion of the statistical analysis. To examine the energy intake and macronutrients, researchers used a 24-h dietary recall on 3 days (one weekend day and two weekdays) at the beginning and the end of the research.

Because of the immorality of removing patients’ drugs, asthma and allergy specialist used the same drugs for all the participants. A code was assigned to each patient to maintain the confidentiality of information.

### 2.1 Participants

The study participants included 40 women and 30 men ranging from 18 to 65 years old. Based on its severity (FEV1 ≥ 60%) and clinical symptoms by the GINA diagnostic criteria, participants were divided into two groups: allergic asthma with mild and moderate (by asthma and allergy specialist). The participants were asked to take the supplements regularly, and how to take them was fully explained. Social information, drugs, lifestyle, and medical history were completed using a questionnaire. Persistent allergic asthma (mild to moderate), body mass index (BMI) less than 30 kg/m^2^, serum IgE ≥30 IU, insensitivity to pomegranate, and age of 18–65 years old were involved in the inclusion criteria of the study.

The exclusion criteria of this study included unwillingness, pregnancy, lactation, smoking, malignancy, taking multivitamin/mineral supplements during the last 2 months, diabetes, autoimmune diseases, and other lung diseases. The current study was recorded in the Iranian Registry of Clinical Trials (IRCT20200205046384N1). Also, the Ahvaz Jundishapur University of Medical Sciences Ethics Committee approved the present trial.

### 2.2 Intervention

The intervention group was given a pomegranate extract capsule containing 250 mg of pomegranate seed extract twice a day (500 mg in total) for 8 weeks. The control group was given placebo capsules (rusk powder). The placebo capsule and the pomegranate extract capsule were similar in terms of size, color, and shape. The placebo and pomegranate extract were provided by Karaj Institute of Medicinal Plants, Alborz Province, Iran. Ghavipour et al. proved that the daily consumption of 500 mg of pomegranate extract for 8 weeks in patients with rheumatoid arthritis reduces inflammation and oxidative stress (Ghavipour et al., 2017). Therefore, in this research, the effect of 500 mg of pomegranate extract was evaluated for 8 weeks. Semi-industrial grinding machines, semi-industrial percolation, vacuum filters, and semi-industrial spray dryers are used to prepare pomegranate extract.

1. Ground sweet pomegranate seeds (powder) and solvent (water) were extracted in a semi-industrial percolation machine at a temperature of 50 °C for 8 h.
2. The liquid extract was filtered by a vacuum filter and concentrated in a spray dryer, and a powder extract was obtained.
3. The powder extract was packed in the form of capsules.

### 2.3 Endpoints

The investigated outcomes included the clinical symptoms of asthma, which were assessed at the beginning and end of the study. The clinical symptoms of asthma consisted of daily breath shortness, nocturnal breath shortness, salbutamol spray usage, nocturnal waking up, and limitation of asthma-related activity.

In addition, the other outcomes were recorded, including basophil, eosinophil, and neutrophil counts at the beginning and end of the study.

### 2.4 Asthma clinical symptoms and severity categorization

As previously mentioned, the severity of asthma was defined based on the FEV_1_ and clinical symptoms. Patients with FEV_1_ ≥ 80% were categorized into the mild group, and patients with FEV_1_ between 60% and 80% were classified into the moderate group. Clinical asthma symptoms were also evaluated at the start and end of the study.

### 2.5 Blood samples

At the start of the research and the end of the eighth week, 6 mL of venous blood was drawn from all patients. After centrifugation, blood samples were kept in the freezer at −80 °C until the end of the study.

### 2.6 Laboratory analyses

The total IgE concentration in patients’ serum was measured using a Roche enzyme-linked immunosorbent assay (ELISA) kit (Germany). CBC-diff was used to examine eosinophils, neutrophils, and basophils. All experiments of this research were performed in the Pasteur laboratory, Ahvaz, Iran.

### 2.7 Dietary analysis and blood pressure

To evaluate the patients’ diet in terms of total calories and received macronutrients, a 24-h dietary recall was used on a one-day weekend and two weekdays at the beginning and the end of the research. Nutritionist 4 (N4) software was used for patients’ diet analysis. Patients’ calorie intake can influence their weight; therefore, their calorie intake was assessed. In this study, a Mercury sphygmomanometer was applied to evaluate blood pressure based on the standard method after 5–10 min of rest at the beginning and the end of the study.

### 2.8 Methods of measuring ellagic acid and punicalagin

Ellagic acid is a thermodynamically extremely stable molecule retaining four rings representing its lipophilic domain. It also has four phenolic groups and two lactone groups, which act as donors and acceptors of hydrogen bonding, respectively (Bala et al., 2006). In this study, a high-performance liquid chromatography (HPLC) method was utilized to measure ellagic acid. Punicalagin is a high-weight molecule that is a polyphenol with antioxidant and water-soluble properties that is extracted from pomegranate fruit (Kulkarni et al., 2007). In addition, the punicalagin was measured by the HPLC method. To evaluate the amounts of bioactive compounds in pomegranate extract, some capsules were randomly analyzed by the Medicinal Plants and Drugs Research Institute of Shahid Beheshti University, Tehran, Iran.

### 2.9 Statistical analysis

Quantitative variables (age, duration of asthma, *etc.*) were reported as mean ± standard deviation, and qualitative variables (gender, the severity of asthma, *etc.*) were reported using numbers (percentage). Data with a non-normal distribution were described as the median (mid-quartile range). The Shapiro–Wilk test was used to assess the normality of quantitative variables. The chi-square test (or Fisher’s exact test) was applied to examine the relationship between qualitative variables (intervention group *versus* control group). In addition, the Mann–Whitney *U* test and independent *T*-test were used for non-parametric and parametric data, respectively. The paired *T*-test and Wilcoxon for parametric and non-parametric variables were respectively applied to evaluate the mean of quantitative variables in each group at the beginning and the end of the study. All research hypotheses were examined at the 5% level using software SPSS 22.

## 3 Results

**FIGURE 1:**
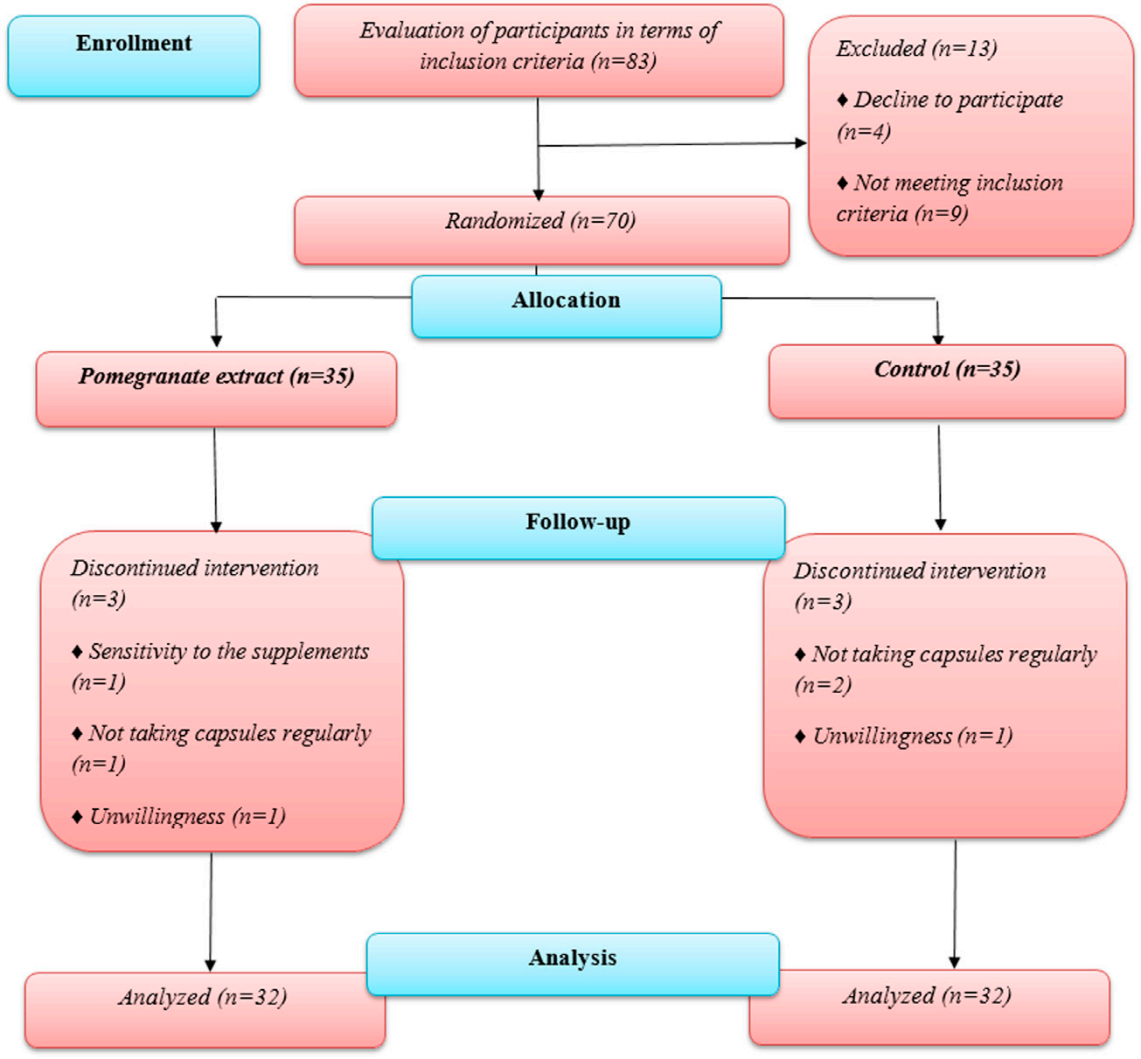
The flowchart of the study.

In this research, 70 people participated, from whom 64 completed the study. Out of 70 people who participated in the study, 64 people finished the research. The reasons for the exclusion of other participants are shown in Figure 1.

### 3.1 Baseline characteristics

In Table 1, the patient’s demographic information is depicted. It can be perceived that there was no significant difference based on dietary variables, demographic features, anthropometric indices, clinical characteristics, asthma severity, physical activity, age of asthma onset, and infant feeding between the control and intervention groups (*p* > 0.05). Also, there was no statistically significant difference between the control and pomegranate extract groups based on the drugs taken by patients (Symbicort, Tiova, Budesonide, and Salbutamol spray) (*p* > 0.05).

**TABLE 1:** Comparison of basic characteristics between intervention and control groups.

Variable	Pomegranate (n = 32)	Control (n = 32)	*p* –value*
Gender			
Male N (%)	15 (46.9)	12 (37.5)	0.307
Female N (%)	17 (53.1)	20 (62.5)	
Age (years)	38.94 ± 13.07	37.94 ± 10.80	0.740
Age of the onset of asthma (year)	28.72 ± 16.23	26.88 ± 16.78	0.651
Asthma severity			
Mild N (%)	16 (50)	16 (50)	0.599
Moderate N (%)	16 (50)	16 (50	
Systolic blood pressure (mm Hg)	120.00 (110.00–125.00)	120.00 (110.00–125.00)	0.916
Diastolic blood pressure (mm Hg)	77.50 (70.00–80.00)	80.00 (70.00–80.00)	0.444
Physical activity (MET-min/week)	2,257.19 ± 315.08	2,126.05 ± 266.13	0.200
Symptoms n (%)			
Daily breath shortness	20 (62.5)	18 (56.3)	0.799
Nocturnal breath shortness	13 (40.6)	12 (37.5)	>0.99
Nocturnal waking up limitation of asthma-related activity	11 (34.4)	8 (25.0)	0.585
limitation of asthma‐related activity	11 (34.4)	16 (50)	0.311
Salbutamol spray consumption	7 (21.9)	5 (15.6)	0.750
Infant feeding N (%)			
Formula	2 (6.2)	2 (6.2)	0.922
Breast feeding	26 (81.3)	27 (84.4)	
Formula + Breast feeding	4 (12.5)	3 (9.4)	

MET: Metabolic Equivalent of Task**.** for parametric data, values have been reported as mean ± standard deviation. For non-parametric data, values have been reported as median (25th, 75th percentiles) and nominal qualitative variables have been described as number (percent).*Reported *p*-value to compare the research variables between the groups: non-parametric and parametric variables were tested using Mann–Whitney *U* test and independent samples *T*-test, respectively. For qualitative variables, the chi-square test and Fisher’s exact test were utilized.

### 3.2 Energy intake, physical activity, anthropometric indices, and blood pressure

Table 2 compares anthropometric indices, physical activity, dietary intake, and blood pressure in two groups of pomegranate extract and control. There was no statistically significant difference in any of the mentioned variables between the two groups at the beginning and the end of the study (*p* > 0.05).

**TABLE 2:** Comparing anthropometric indices, physical activity, dietary intake, and blood pressure in the intervention and control groups.

Variable	Pomegranate (n = 32)	Control (n = 32)	*p*-value*
Height (cm)	167.75 ± 10.65	162.72 ± 8.54	0.078
Weight (kg)			
Baseline	73.90 ± 14.43	72.25 ± 11.23	0.610
Endpoint (8 weeks)	74.46 ± 14.63	72.39 ± 11.45	0.529
Changes	0.00 (−1) to (1)	0.00 (−1.75) to (1)	0.897
*p*-value**	0.293	0.789	
Waist circumference (cm)			
Baseline	89.41 ± 13.33	89.66 ± 12.19	0.938
Endpoint (8 weeks)	89.75 ± 13.12	89.41 ± 12.18	0.914
Changes	0.00 (−0.75) to (0.00)	0.00 (−1.75) to (1.75)	0.868
*p*-value**	0.498	0.667	
Hip circumference (cm)			
Baseline	104.03 ± 9.64	104.75 ± 9.21	0.762
Endpoint (8 weeks)	103.94 ± 10.03	105.01 ± 8.82	0.654
Changes	0.00 (0.00) to (0.00)	0.00 (0.00) to (0.00)	0.276
*p*-value**	0.693	0.325	
WHR			
Baseline	0.85 ± 0.07	0.84 ± 0.06	0.488
Endpoint (8 weeks)	0.85 ± 0.08	0.84 ± 0.07	0.519
Changes	0.00 (0.00) to (0.00)	0.00 (−0.01) to (0.01)	0.673
*p*-value**	0.872	0.872	
Physical activity (MET-min/week)			
Baseline	2,257.19 ± 315.08	2,126.05 ± 266.13	0.200
Endpoint (8 weeks)	233.06 ± 571.46	2,127.69 ± 235.01	0.115
Changes	−77.50 (−145.00) to (153.37)	−70.00 (−151.75) to (146.25)	0.773
*p*-value**	0.378	0.970	
Energy (kcal/day)			
Baseline	2090.32 ± 844.27	2073.72 ± 812.28	0.936
Endpoint (8 weeks)	2,323.38 ± 739.09	2,118.38 ± 848.81	0.307
Changes	145.00 (−152.75) to (747.00)	48.50 (−300.00) to (392.25)	0.379
*p*-value**	0.114	0.700	
Percentage of calories from carbohydrate (%)			
Baseline	58.99 ± 9.46	57.46 ± 10.25	0.538
Endpoint (8 weeks)	61.32 ± 10.22	57.47 ± 9.97	0.134
Changes	−1.00 (−4.75) to (6.65)	−1.75 (−3.90) to (5.00)	0.648
*p*-value**	0.186	0.996	
Percentage of calories from protein (%)			
Baseline	12.88 ± 3.24	14.88 ± 5.91	0.310
Endpoint (8 weeks)	13.88 ± 3.05	13.75 ± 4.16	0.908
Changes	0.85 (0.00) to (3.30)	−1.00 (−4.00) to (3.95)	0.070
*p*-value**	0.099	0.351	
Percentage of calories from fat (%)			
Baseline	28.24 ± 8.25	27.68 ± 8.27	0.786
Endpoint (8 weeks)	26.25 ± 10.60	28.69 ± 8.74	0.340
Changes	0.00 (−4.62) to (3.30)	1.50 (−4.00) to (4.77)	0.432
*p*-value**	0.251	0.419	
SBP (mm Hg)			
Baseline	120.00 (110.00–125.00)	120.00 (110.00–125.00)	0.916
Endpoint (8 weeks)	120.00 (110.00–120.00)	120.00 (110.00–125.00)	0.454
Changes	0.00 (−10.00) to (5)	0.00 (−3.75) to (0.00)	0.723
*p*-value**	0.191	0.170	
DBP (mm Hg)			
Baseline	77.50 (70.00–80.00)	80.00 (70.00–80.00)	0.444
Endpoint (8 weeks)	70.00 (70.00–80.00)	80.00 (70.00–80.00)	0.315
Changes	0.00 (−8.75) to (0.00)	0.00 (−3.75) to (3.75)	0.288
*p*-value**	0.353	0.754	

MET: metabolic equivalent of task, SBP: systolic blood pressure, DBP: diastolic blood pressure, BMI: body mass index, WHR: waist to hip ratio, * Reported *p*-value to compare the research variables between the groups: independent samples *T*-test and Mann–Whitney *U* test were used for parametric and non-parametric variables, respectively, ** Reported *p*-value to evaluate the research variables within the groups (paired *T*-test and Wilcoxon test for parametric and non-parametric variables, respectively). Values have been reported as median (25th, 75th percentiles) and mean ± standard deviation for non-parametric and parametric data, respectively.

### 3.3 Asthma symptoms

At the beginning of the study, there was no statistically significant difference in terms of asthma symptoms and salbutamol spray consumption. At the end of the study, the findings indicated that supplementation with pomegranate extract improved day-and-night shortness of breath and activity limitation due to asthma symptoms in the intervention group *versus* the control group (*p* < 0.05). There was no observed a statistically significant difference in terms of salbutamol spray consumption at the end of the study (*p* > 0.05) (Table 3; Table 4).

**TABLE 3:** Comparing clinical changes, basophil, eosinophil, and neutrophil counts between the intervention and control groups.

Symptoms	Pomegranate (n = 32)	Control (n = 32)	*p*-value^a^
Daily breath shortness; n (%)			
Baseline	20 (62.5)	18 (56.3)	0.799
Endpoint (8 weeks)	7 (21.9)	21 (65.6)	0.001
*p*-value**	<0.0001	0.607	
Nocturnal breath shortness; n (%)			
Baseline	13 (40.6)	12 (37.5)	>0.99
Endpoint (8 weeks)	2 (6.3)	12 (37.5)	0.005
*p*-value**	0.002	>0.99	
Nocturnal waking up; n (%)			
Baseline	11 (34.4)	8 (25.0)	0.585
Endpoint (8 weeks)	3 (9.4)	7 (21.9)	0.302
*p*-value**	0.008	>0.99	
Limitation of asthma-related activity symptoms; n (%)			
Baseline	11 (34.4)	16 (50)	0.311
Endpoint (8 weeks)	4 (12.5)	14 (43.8)	0.003
*p*-value**	0.016	0.625	
Consumption of salbutamol spray; n (%)			
Baseline	7 (21.9)	5 (15.6)	0.750
Endpoint (8 weeks)	5 (15.6)	3 (9.4)	0.708
*p*-value**	0.625	0.500	
Neutrophil (%)			
Baseline	59.59 ± 7.04	58.48 ± 8.02	0.914
Endpoint (8 weeks)	54.80 ± 6.54	60.73 ± 5.64	<0.0001
Changes	−4.78 ± 5.26	2.24 ± 7.57	<0.0001
*p*-value**	<0.0001	0.103	
Basophil (%)			
Baseline	0.40 (0.30–0.47)	0.40 (0.30–0.50)	0.649
Endpoint (8 weeks)	0.30 (0.22–0.40)	0.30 (0.20–0.40)	0.177
Changes	−0.10 (−0.10) to (0.00)	−0.10 (−0.20) to (0.10)	0.853
*p*-value**	0.005	0.132	
Eosinophil (%)			
Baseline	2.45 (1.30–5.37)	1.05 (0.80–2.57)	0.002
Endpoint (8 weeks)	1.30 (0.80–2.70)	1.70 (0.82–2.95)	0.121^£^
Changes	−0.90 (−2.50) to (−0.10)	0.05 (−0.60) to (1.47)	0.002
*p*-value**	0.001	0.370	

^a^ Reported *p*-value to evaluate the research variables between the groups: independent samples *T*-test and Mann–Whitney *U* test were used for parametric and non-parametric data, respectively. ** *p*-value to compare the within-group variables (paired *T*-test and Wilcoxon test for parametric and non-parametric variables, respectively).

^£^ Using ANCOVA test and adjustment for baseline value. Values have been expressed as median (25th, 75th percentiles) and mean ± standard deviation for non-parametric and parametric data, respectively.

**TABLE 4:** Comparing clinical changes between the intervention and control groups.

Symptoms (frequency per day)	Pomegranate (n = 32)	Placebo (n = 32)	*p*-value^a^
Daily breath shortness			
Baseline	2.00 (0.00–3.00)	1.00 (0.00–2.00)	0.198
Endpoint (8 weeks)	0.00 (0.00–0.00)	1.00 (0.00–2.00)	0.001
Changes	−1.00 (−3.00) to (0.00)	0.00 (−1.00) to (1.00)	<0.001
*p*-value**	<0.001	0.791	
Nocturnal breath shortness			
Baseline	0.00 (0.00–1.00)	0.00 (0.00–1.75)	0.921
Endpoint (8 weeks)	0.00 (0.00–0.00)	0.00 (0.00–2.00)	0.003
Changes	0.00 (−1.00) to (0.00)	0.00 (0.00) to (0.00)	0.034
*p*-value**	0.002	0.821	
Nocturnal waking up Baseline	0.00 (0.00–1.00)	0.00 (0.00–0.75)	0.400
Endpoint (8 weeks)	0.00 (0.00–0.00)	0.00 (0.00–0.00)	0.188
Changes	0.00 (−1.00) to (0.00)	0.00 (0.00) to (0.00)	0.028
*p*-value**	0.002	0.834	
Limitation of asthma-related activity symptoms			
Baseline	0.00 (0.00–1.00)	0.50 (0.00–2.00)	0.222
Endpoint (8 weeks)	0.00 (0.00–0.00)	0.00 (0.00–2.75)	0.003
Changes	0.00 (−1.00) to (0.00)	0.00 (0.00) to (0.00)	0.032
*p*-value**	0.003	0.991	
Consumption of salbutamol spray			
Baseline	0.00 (0.00–0.00)	0.00 (0.00–0.00)	0.601
Endpoint (8 weeks)	0.00 (0.00–0.00)	0.00 (0.00–0.00)	0.536
Changes	0.00 (0.00) to (0.00)	0.00 (0.00) to (0.00)	0.222
*p*-value**	0.172	0.280	

^a^ Mann–Whitney *U* test ** Wilcoxon test. Values have been expressed as median (25th, 75th percentiles).

### 3.4 Neutrophil, basophil, and eosinophil counts

Mean blood levels of neutrophils, basophils, and eosinophils in blood cell count were compared between the control and intervention groups at the start and the end of the study. In terms of basophils and neutrophils, no statistically significant difference was found between the intervention group and the control group at the beginning of the study (*p* > 0.05). Still, eosinophils had a statistically significant difference (*p* = 0.002). The findings showed that within-group differences were statistically significant in eosinophils, neutrophils and basophils in the group receiving pomegranate extract (*p* = 0.001, *p* < 0.0001 and *p* = 0.005, respectively). Also, the results indicated that supplementation with pomegranate extract significantly reduced neutrophils in the intervention group compared to the control group (*p* < 0.0001). At the end of the study, no statistically significant difference was observed between the intervention group and the control group at the end of the study in eosinophils (*p* = 0.121). Furthermore, the change levels in neutrophils and eosinophils in the intervention group compared to the control group were statistically significant at the end of the study (*p* < 0.0001 and *p* = 0.002, respectively). In terms of basophils, no statistically significant difference was detected between the intervention group and the control group at the end of the study (*p* > 0.05) (Table 3).

### 3.5 Ellagic aid and punicalagin alpha and beta

The ellagic acid and punicalagin were measured by the HPLC method. Each 250 mg capsule of pomegranate extract contained 2.1 μg of ellagic acid, 118.4 μg of punicalagin alpha, and 53 μg of punicalagin beta.

## 4 Discussion

The current study used a randomized controlled trial that was performed employing a double-blind design in which the effects of pomegranate (500 mg/day for 8 weeks) in persistent allergic asthma were investigated in terms of clinical symptoms and CBC-diff. The findings showed that pomegranate extract could improve daily breath shortness, nocturnal breath shortness, and limitation of asthma-related activity and can also decrease neutrophils and eosinophils in the pomegranate receiving group in comparison to the control group. Since this research was the first one to investigate the effects of pomegranate extract on allergic asthma, this has made it difficult to evaluate the results of our research with similar studies. In a study conducted on the impact of saffron on the clinical symptoms in patients with allergic asthma, the results showed that it could improve the clinical symptoms (Zilaee et al., 2019) because saffron, like pomegranate, has anti-inflammatory and antioxidant properties.

The results revealed that pomegranate extract supplementation exerted a statistically significant effect on reducing the neutrophil, basophil, and eosinophil counts. Moreover, pomegranate extract significantly decreased neutrophil and eosinophil counts in the intervention group compared to the control group. Inflammation, increased airway response, and structural changes are the main features of asthma. Eosinophils represent the primary cells involved in inflammation and asthma pathology. In non-allergic and allergic asthma, these cells’ development, maturity, activation, and survival influence the respiratory system (Pelaia et al., 2015). Not only eosinophil-induced inflammation but also neutrophil-induced inflammation is involved in the pathogenesis of asthma. They cause the secretion of a wide range of products, including leukotrienes, cytokines, proteases, and metalloproteins. Also, neutrophil products cause airway obstruction, excessive mucus secretion, and airway over-response (Bloemen et al., 2007). This inflammatory process leads to shortness of breath, coughing, and wheezing. Therefore, the elimination of inflammation in the airways occurs naturally in the host body as an active reaction (Barnig et al., 2018). In the present study, the improvement in the clinical symptoms of the patients could be justified by the decrease in eosinophil and neutrophil counts. Also, improvement in the clinical symptoms of patients led to improvement in lung function parameters (using spirometry tests) (Shateri et al., 2022).

Studies have shown that pomegranate extract reduces inflammatory mediators in different ways, but there is little information about the specific anti-allergic property of pomegranate (Ismail et al., 2012). According to the studies, ellagitannin and ellagic acid represent polyphenols found in some fruits, including pomegranate, are rich in anti-inflammatory, antioxidant and anti-cancer properties. Over the past few years, pomegranate has received much attention as a new treatment approach (Danesi and Ferguson, 2017). Furthermore, punicalagin maintains the highest antioxidant effect compared to the other pomegranate polyphenols. Punicalagin is a complex ellagitannin responsible for more than 50% of pomegranate’s antioxidant activity (Bayat-Chadegani et al., 2015). In addition, based on previous studies conducted on animal models, it has been proven that ellagic acid has an apparent anti-inflammatory effect by inhibiting nuclear factor kappa-B (NF-KB) activation in mice with asthma. So, it can be considered a therapeutic agent for allergic asthma (Zhou et al., 2014).

Also, the current study showed that supplementation with pomegranate extract in asthma patients compared with the control group did not cause any remarkable difference in systolic and diastolic blood pressure. There are inconsistent findings on the impact of pomegranate on blood pressure. Some studies supported the influence of pomegranate on systolic and diastolic blood pressure (Sohrab et al., 2008; Giménez-Bastida et al., 2021). Still, other studies did not support its effectiveness (Esmaillzadeh et al., 2004; Sumner et al., 2005). Various mechanisms have been suggested for the impact of the pomegranate on blood pressure. It has been shown that vitamin C can effectively reduce systolic and diastolic blood pressure by improving and restoring nitric oxide activity through vasodilation of the arteries (Shateri et al., 2016). Pomegranate also contains different amounts of vitamin C in the range of 52.8 mg–72 mg/100 g of fresh pomegranate seeds (Opara et al., 2009). In addition, one of the mechanisms of the pomegranate on blood pressure is its inhibitory effect on the angiotensin-converting enzyme (Sohrab et al., 2008). The possible reason for inconsistent systolic and diastolic blood pressure results in the present study *versus* previous studies could be caused by the differences in the sample size, population, and the different parts of consumed pomegranate.

This research, like many other studies, contains its limitations. We employed self-report tools to assess asthma symptoms in this study, so participants may not have provided accurate information. In addition, if this intervention is performed for a more prolonged period, more reliable results are likely to be obtained. The strengths of the current study included a double-blind, randomized, placebo-controlled scheme and the first clinical trial to assess the impacts of pomegranate extract in a human model of allergic asthma.

## 5 Conclusion

The pomegranate extract seems to decrease neutrophil, basophil, and eosinophil in patients with allergic asthma and improve some of their clinical symptoms. Although the effect of pomegranate extract on asthma has been examined for the first time in the human model, studies with more sample sizes and more extensive periods should be conducted to confirm the results and effectiveness of pomegranate extract in clinical settings.

## Data Availability

The raw data supporting the conclusion of this article will be made available by the authors, without undue reservation.
